# Flagellar brake protein YcgR interacts with motor proteins MotA and FliG to regulate the flagellar rotation speed and direction

**DOI:** 10.3389/fmicb.2023.1159974

**Published:** 2023-04-14

**Authors:** Qun Han, Shao-Feng Wang, Xin-Xin Qian, Lu Guo, Yi-Feng Shi, Rui He, Jun-Hua Yuan, Yan-Jie Hou, De-Feng Li

**Affiliations:** ^1^State Key Laboratory of Microbial Resources, Institute of Microbiology, Chinese Academy of Sciences, Beijing, China; ^2^College of Life Sciences, University of Chinese Academy of Sciences, Beijing, China; ^3^Hefei National Laboratory for Physical Sciences at the Microscale and Department of Physics, University of Science and Technology of China, Hefei, Anhui, China; ^4^National Laboratory of Biomacromolecules, CAS Center for Excellence in Biomacromolecules, Institute of Biophysics, Chinese Academy of Sciences, Beijing, China

**Keywords:** c-di-GMP, YcgR, flagellar brake protein, flagellar motility, *Escherichia coli*

## Abstract

In *E. coli* and related species, flagellar brake protein YcgR responds to the elevated intracellular c-di-GMP, decreases the flagellar rotation speed, causes a CCW rotation bias, and regulates bacterial swimming. Boehm et al. suggested that c-di-GMP-activated YcgR directly interacted with the motor protein MotA to curb flagellar motor output. Paul et al. proposed that YcgR disrupted the organization of the FliG C-terminal domain to bias the flagellar rotation. The target proteins are controversial, and the role of motor proteins remains unclear in flagellar rotation speed and direction regulation by YcgR. Here we assayed the motor proteins’ affinity via a modified FRET biosensor and accessed the role of those key residue via bead assays. We found that YcgR could interact with both MotA and FliG, and the affinities could be enhanced upon c-di-GMP binding. Furthermore, residue D54 of YcgR-N was needed for FliG binding. The mutation of the FliG binding residue D54 or the MotA binding ones, F117 and E232, restored flagellar rotation speed in wild-type cells and cells lacking chemotaxis response regulator CheY that switched the flagellar rotation direction and decreased the CCW ratio in wild-type cells. We propose that c-di-GMP-activated YcgR regulated the flagellar rotation speed and direction via its interaction with motor proteins MotA and FliG. Our work suggest the role of YcgR-motor proteins interaction in bacterial swimming regulation.

## Introduction

The transition between motile and sessile is essential for bacterial survival (Mahto et al., 2022). To adapt to various environments, many kinds of bacteria can migrate toward favorable conditions and form biofilm attached to surfaces, along with the capability of switching between the free-swimming style and the surface-attached fashion (O'Toole et al., 2000; Donlan, 2002). The mechanism of bacteria regulating the lifestyle switch has been explored in recent decades. Cyclic di-GMP (c-di-GMP) is identified as a universal second messenger across prokaryotes, including Proteobacteria and Firmicutes, and is extensively involved in biofilm formation and dispersal (Fagerlund et al., 2016; Wang et al., 2018; Yang et al., 2018; Liu et al., 2022). It could achieve motility regulation by controlling the expression of flagella and chemoreceptor genes or directly targeting the flagella. Bacterial motility is well exemplified by *Escherichia coli*, which is a classic model for studying bacterial swimming and chemotaxis. In response to disadvantageous environmental cues, bacteria could upregulate the synthesis of c-di-GMP, downregulate the expressions of those flagellar protein genes, capsulize themselves with extracellular polymeric substances (EPS), and shift a free-swimming style to a surface-attached one (Macnab, 1992; Jenal et al., 2017; Laventie and Jenal, 2020; Suchanek et al., 2020). This kind of regulation is usually found in Proteobacteria and Firmicutes. Besides, the posttranscriptional regulation of bacterial swimming was reported for *E. coli* and related species in a c-di-GMP-dependent manner *via* flagellar brake protein YcgR, a PilZ domain-containing protein that binds c-di-GMP (Armitage and Berry, 2010; Boehm et al., 2010; Paul et al., 2010; Zorraquino et al., 2012; Subramanian et al., 2017).

The swimming of *E. coli* is propelled by the rotation of flagella, a nano-machine composed of various proteins (Tan et al., 2021; Hu et al., 2022; Mondino et al., 2022). The core structure of flagella is the motor complex which consists of a rotating part (the rotor) and a membrane-embedded non-rotating part (the stator; Paul et al., 2010; Nakamura and Minamino, 2019; Zhuang and Lo, 2020). The rotor is composed of dozens of copies of FliG (~34) and FliM (~34) and more than 100 copies of FliN with a FliG/FliM/FliN ratio of 1:1:3 (Carroll et al., 2020; Tan et al., 2021) and involved in flagellar rotation and direction control. The stator complex is formed from five copies of MotA and two copies of MotB (Deme et al., 2020; Santiveri et al., 2020; Hu et al., 2022), conducts ions across the membrane, and generates the torque to drive the flagellar rotation *via* the electrostatic interactions between the stator protein MotA and the rotor protein FliG (Morimoto et al., 2013; Nakamura et al., 2014; Takekawa et al., 2014; Subramanian et al., 2017).

*Escherichia coli* cells are proposed to elevate its intracellular c-di-GMP concentration at specific conditions, such as starvation, stationary growth conditions, or the deletion of a phosphodiesterase gene *yhjH*, and then recruit YcgR to reduce bacterial swimming speed (Boehm et al., 2010; Paul et al., 2010; Le Guyon et al., 2015; Kojima et al., 2019). This regulation confers the cells the ability to respond to environmental stimuli rapidly and makes it possible for motile bacteria to attach to a surface and initiate biofilm formation. For example, the deletion of gene *yhjH* was observed to increase the intracellular c-di-GMP concentration and inhibit the swimming of *E. coli*. Different groups have studied the mechanism of how c-di-GMP worked in this situation. It was proposed that the elevated c-di-GMP levels resulted in YcgR binding to c-di-GMP. The c-di-GMP-bound YcgR was identified to suppress the flagellar movement by directly interacting with the motor protein(s). Consequently, it decreased the flagellar rotation speed and caused a CCW rotation bias. Nieto et al. proved that the bias of flagellar rotation and the reduction of motor output sequentially occurred upon the induction of YcgR expression (Wang et al., 2018; Nieto et al., 2019).

However, the target proteins of YcgR and the mechanism of how YcgR functions proposed by Boehm et al. and Paul et al. are controversial. Boehm et al. showed that activated YcgR interacted with MotA *via in vivo* FRET assay and suggested that c-di-GMP-activated YcgR inhibited motility by directly interacting with the motor protein MotA to curb flagellar motor output (Boehm et al., 2010). Paul et al. observed that YcgR interacted with the flagella switch-complex proteins FliG and FliM most strongly in the presence of c-di-GMP *via* pull-down and two-hybrid assays (Paul et al., 2010). They proposed that YcgR disrupted the organization of the FliG C-terminal domain, which interacts with the stator protein MotA to generate torque, biased the flagellar rotation, and reduced the motor output (Armitage and Berry, 2010). Fang and Gomelsky showed the interaction of YcgR and FliG and YcgR mutant R118D and FliM *via* pull-down and two-hybrid assays. They suggested that YcgR altered the interaction of FliG and FliM and regulated the flagellar rotation (Fang and Gomelsky, 2010). In our previous study, gel filtration and SAXS assays showed that activated-YcgR interacted with MotA *via* its PilZ domain. The YcgR-N domain, independent of MotA interaction, is also necessary for motility regulation, but its role still needs to be explored (Hou et al., 2020). More importantly, it still lacks the YcgR mutagenesis evidence to indicate whether and how motor proteins interact with YcgR to regulate the output of a single motor.

Here, we used a modified FRET method to determine the affinities between YcgR and motor proteins and identified the FliG binding site. *Via* mutagenesis assays, we found that both YcgR-MotA and YcgR-FliG interactions decreased the flagellar rotation speed and biased the rotation direction in the presence of elevated c-di-GMP concentration. Our work provided more knowledge to solve the puzzle of how YcgR regulated flagellar swimming.

## Results

### A modified FRET assay was established to determine the motor proteins’ affinities

To examine precisely to which proteins YcgR binds and how the motor proteins’ affinities of YcgR are, we used a modified *in vitro* fluorescence resonance energy transfer (FRET) assay in this research. A previous study constructed a FRET-based YcgR biosensor by fusing YcgR from *Salmonella typhimurium* between N-terminal yellow and C-terminal cyan fluorescence proteins (YFP and CFP; Christen et al., 2010). The FRET property of the biosensor, which depended on the two fluorescence subunits’ relative orientation, was represented by the FRET/CFP emission ratio, as suggested by Christen et al. (2010). It was proposed that the c-di-GMP binding altered the relative orientation of CFP and YFP. The biosensor was used to monitor c-di-GMP concentrations *via* FRET/CFP ratio. The *E. coli* and *S. Typhimurium* YcgR share about 73% sequence identity. The *S. Typhimurium* YcgR sequence in that FRET-based YcgR biosensor was replaced by the *E. coli* one to construct our biosensor.

**Figure 1:**
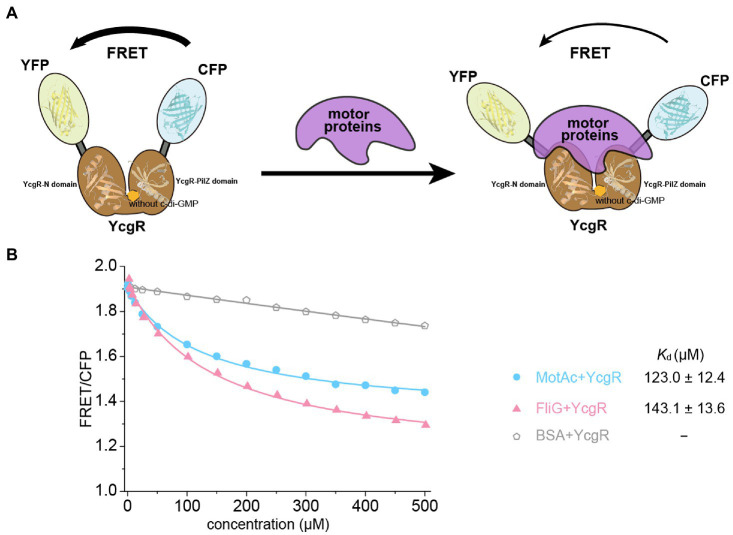
YcgR bound to motor proteins in the FRET assay. **(A)** Scheme illustrating how binding of YcgR to motor proteins leads to a detectable change in FRET between attached yellow (YEP) and cyan (CEP) fluorescent protein domains. The fluorescent subunits were nearby without motor proteins, and FRET was maximal. Motor protein binding to YcgR induced a conformational change that separated the fluorescent domains, decreasing FRET efficiency. **(B)** Profile of the FRET/CFP emission fluorescence ratio along the increasing concentration of motor proteins (blue line for MotAc and red for FliG). The *K*d (μM) between YcgR and motor proteins were calculated accordingly and shown as mean ± SE. The experiments were repeated three times, and representative examples were shown.

In an *in vitro* assay, the YcgR biosensor was mixed with purified motor proteins. In the absence of c-di-GMP, we found that the FRET/CFP ratio of the YcgR biosensor was gradually decreased by the elevated concentrations of the cytoplasmic domain of MotA (residues 70–170 of MotA), MotAc (Zhou et al., 1995) and individual FliG (Figure 1; Supplementary Figure S1). The results indicate that the motor proteins could alter the relative orientation of CFP and YFP subunits of the YcgR biosensor. We proposed that motor proteins may occupy the position where previously accommodated CFP or YFP in the YcgR biosensor, induced a domain rearrange of CFP and YFP, and then altered the FRET efficiency (Figure 1A). Compared with the negative control where BSA protein was used, the FRET/CFP ratio plots suggest that YcgR interacted with the tested motor proteins MotAc and FliG without c-di-GMP. The ratios were then fitted into a logistic function, and the dissociation constants (*K*_d_) of YcgR interacting with MotAc or FliG were calculated as 123.0 ± 12.4 and 143.1 ± 13.6 μM, respectively (Figure 1B). The affinities between YcgR and motor proteins were relatively low, consistent with the observation that ligand-free YcgR did not form a stable complex with MotAc or FliG in gel filtration (Hou et al., 2020).

### The c-di-GMP binding increased the motor proteins’ binding ability of YcgR

**Figure 2:**
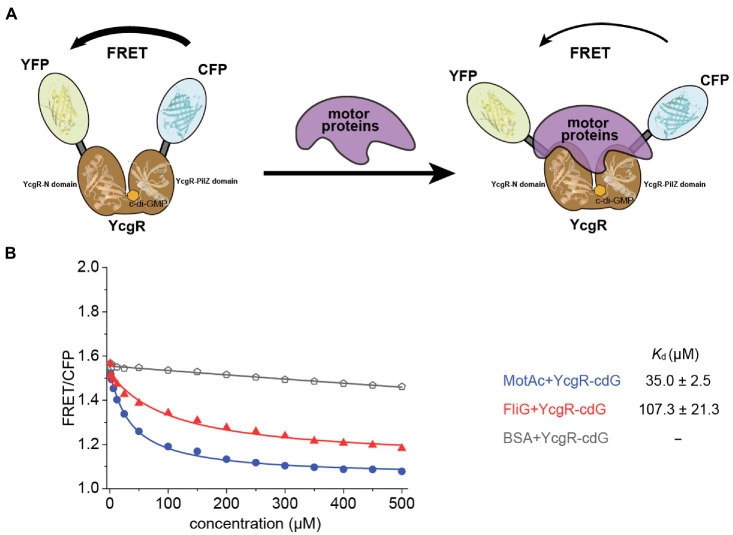
The binding of c-di-GMP increased the motor proteins’ affinities of YcgR. **(A)** Scheme illustrating the detectable change in FRET caused by binding of YcgR oversaturated with c-di-GMP (cdG) to motor proteins. The YcgR-c-di-GMP complex, in the absence of motor proteins, possessed close fluorescent subunits, and FRET is maximal. Motor protein binding induced a conformational change separating the fluorescent domains, decreasing FRET efficiency. The overlap of motor proteins and c-di-GMP in the diagram did not mean motor proteins bound to c-di-GMP. Instead, the MotA binding site was involved in one c-di-GMP binding motif. **(B)** Profile of the FRET/CFP emission fluorescence ratio along the increasing concentration of motor proteins (blue line for MotAc and red line for FliG). The *K*d (μM) between YcgR and motor proteins were calculated accordingly and shown as mean ± SE. The experiments were repeated three times, and representative examples were shown.

YcgR bound c-di-GMP and regulated flagellar motility in the elevated cellular c-di-GMP concentration. Thus, the target proteins of c-di-GMP-bound YcgR are the key to revealing the mechanism of how YcgR inhibited flagellar motility. We attempted to determine the motor proteins’ affinities of c-di-GMP-bound YcgR *via* the above FRET assay. Since both c-di-GMP and motor proteins altered the relative orientation of CFP and YFP, the excess c-di-GMP (2 mM) was used in the FRET assay to oversaturate the YcgR biosensor (1.5 μM). The dissociation constant of YcgR to c-di-GMP was ~0.14 μM (Hou et al., 2020), and we concluded that almost all YcgR biosensors (>99%) had bound c-di-GMP under those concentrations. Assuming that YcgR bound c-di-GMP with a weak affinity (*K*_d_ of 100 μM), we concluded that ~95% of YcgR still bind c-di-GMP under the above c-di-GMP concentrations (Hou et al., 2020). Therefore, the FRET assay we used would be unaffected by the possible c-di-GMP affinity variation (Figure 2A).

FRET/CFP data were measured in the presence of gradient concentrations of motor proteins and excess c-di-GMP and then fitted into a logistic equation to calculate the dissociation constant *K_d_*. The *K_d_* for MotAc and FliG were 35.9 ± 2.9, and 107.3 ± 21.3 μM, respectively (Figure 2B; Supplementary Figure S1). The data showed that c-di-GMP binding significantly increased the MotAc affinity of YcgR from *K*_d_ of 123.0–35.9 μM, whereas the FliG affinities were slightly shifted by c-di-GMP (*K*_d_ of 143.1–107.3 μM). A relatively high MotAc affinity of c-di-GMP-bound YcgR (*K_d_* of 35.9 μM) agreed that only MotAc and c-di-GMP-bound YcgR formed a stable complex in SEC assay (Hou et al., 2020). In summary, YcgR could interact with both stator protein MotA and rotor proteins FliG, and the affinities of both motor proteins could be enhanced by c-di-GMP binding.

### The conserved residue D54 in the YcgR-N domain was involved in the FliG binding to c-di-GMP-bound YcgR

**Figure 3:**
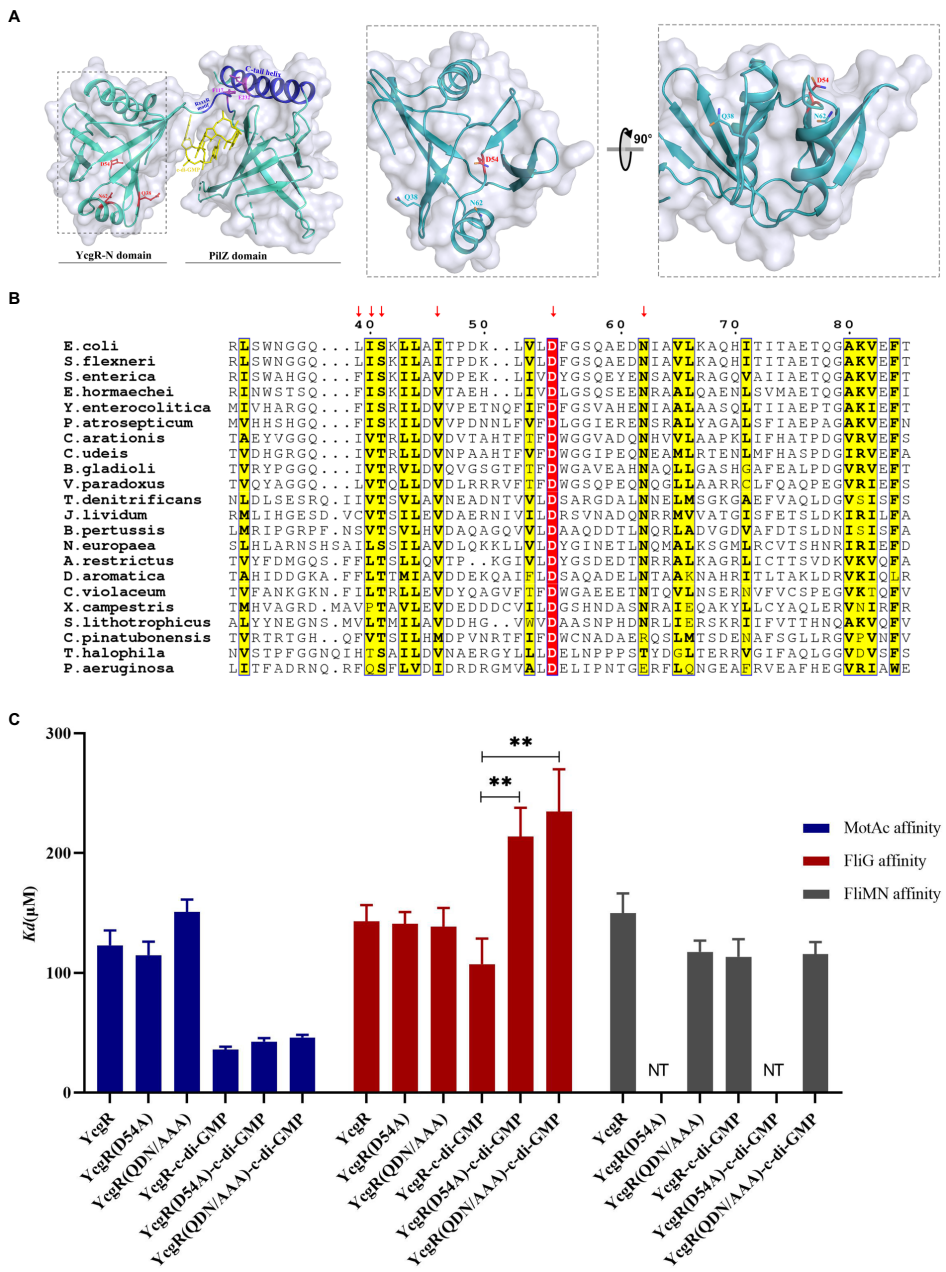
Residue D54 of YcgR was involved in FliG binding. **(A)** The structure of the YcgR protein (derived from PDB 5Y6G) comprised the PliZ domain and the YcgR-N domain. The panel in the dashed line box displayed the conserved residues in the YcgR-N domain. The previously identified MotA binding site was highlighted in blue. **(B)** Sequence alignment of YcgR and similarities from *Escherichia coli* and other 27 prokaryote species. The yellow columns indicated the conserved amino acid residues of YcgR and similarities, including Q38, N62, I40, and L44. The red column indicated residue D54 as the only identical amino acid among those YcgR similarities in the YcgR-N domain. **(C)** The histogram showed the dissociation constants between YcgR variants and motor proteins (MotAc in blue, FliG in red, FliMN in gray) with or without c-di-GMP. NT, not tested. The results were analyzed by statistics method *t*-tests (and nonparametric tests). Analysis of significant differences (Two-tailed):, *p* ≤ 0.01: **.

YcgR contains an N-terminal YcgR-N domain, a C-terminal PilZ domain, and a loop that connects those two domains and contributes to c-di-GMP binding (Hou et al., 2020). Both domains possessed the β-barrel fold clamped by two helices in the YcgR-N domain or situated by one helix over the top in the PilZ domain (Figure 3A). The previous study described how the PilZ domain bound to c-di-GMP and MotA, yet what and how the YcgR-N domain binds to still lack enough evidence. Three hydrophilic residues, Q38, D54, and N62, and two hydrophobic residues, I40 and L44, in the N-terminal YcgR-N domain were revealed as conserved *via* sequence alignment of YcgR and similarities, located at the same side of the YcgR-N domain. They constituted a potential binding site for some unidentified targets (Hou et al., 2020). Residue D54 is the only identical one in the YcgR-N domain among those YcgR similarities (Figure 3B). The mutation of D54A had the maximum swimming ability in those single site-directed mutations involved in the YcgR-N domain in the previous study, and the mutation of Q38A/D54A/N62A (QDN/AAA) did much more than D54A (Hou et al., 2020). The interaction between YcgR biosensor mutants QDN/AAA or D54A and motor proteins was subsequently assayed by FRET.

In the absence of c-di-GMP, the QND/AAA biosensor bound to MotAc and FliG with *K*_d_ of 151.1 ± 10.2 μM and 138.6 ± 15.6 μM, respectively (Figure 3; Supplementary Figure S2A). These disassociation constants (*Kd*) were comparable to those of c-di-GMP-free wild-type YcgR. The D54A mutation also did not severely alter the MotA or FliG affinity (*K*_d_ of 114 ± 11.3 μM and 141.0 ± 9.8 μM) in the absence of c-di-GMP (Figure 3; Supplementary Figure S2B). It was therefore concluded that residues Q38, D54, or N62 were not involved in MotAc or FliG binding in the absence of c-di-GMP.

In the presence of c-di-GMP, the QND/AAA biosensor bound to MotAc with *K*_d_ of 46.1 ± 2.2 μM (Figure 3; Supplementary Figure S1A). The disassociation constant was comparable to that of c-di-GMP-bound wild-type YcgR. The D54A mutation slightly altered the MotA affinity of c-di-GMP-bound YcgR (Figure 3; Supplementary Figure S1B). It was concluded that residues Q38, D54, or N62 were not involved in the MotAc binding to c-di-GMP-activated YcgR. Yet the QND/AAA biosensor showed a significantly lower FliG affinity (*K*_d_ of 234.6 ± 35.4 μM) than the wild-type one (*K*_d_ of 107.3 μM) in the presence of c-di-GMP (Figure 3; Supplementary Figure S1A). Further, the D54A mutation also weakened the FliG affinity of YcgR in the presence of c-di-GMP (*K*_d_ of 213 μM, Figure 3; Supplementary Figure S1B). These results indicated that the conserved residue D54, possibly with those in-vicinity residues, was essential for FliG binding to c-di-GMP-activated YcgR.

### Both YcgR-MotA and YcgR-FliG interactions were involved in regulating flagellar rotation speed and direction

**Figure 4:**
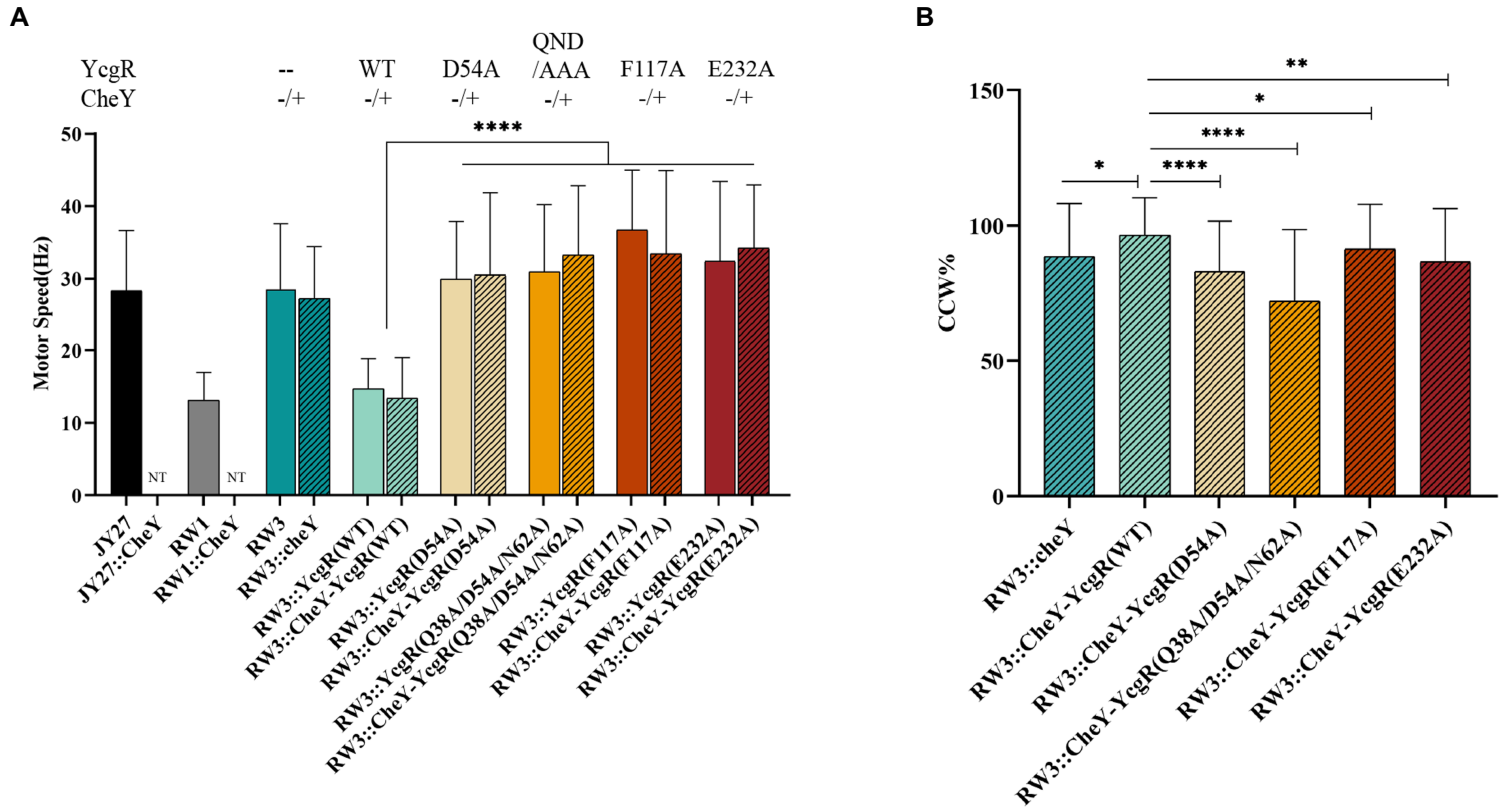
The YcgR-MotA and YcgR-FliG interactions were involved in motility regulation. **(A)** The flagellar rotation speeds of RW3 cells harboring YcgR variants (columns filled with color, the numbers of motors were 23, 47, 24, 28, 31, 24, 40, and 40) and those harboring both cheY and YcgR variants (column filled with lines, the numbers of motors were 44, 52, 45, 38, 45, and 45). **(B)** The CCW ratios of RW3 cells harboring both cheY and YcgR variants. NT, not tested. The speeds and CCW ratios were shown as mean ± SD. The results were analyzed by statistics method *t*-tests (and nonparametric tests). Analysis of significant differences (Two-tailed): *p* ≤ 0.05: *, *p* ≤ 0.01: **, *p* ≤ 0.0001: ****.

YcgR was involved in both the inhibition of flagellar rotation speed and bias of rotation direction. We attempted to tease the relationship between rotation speed and direction altered by YcgR-FliG and YcgR-MotA interaction. *Escherichia coli* JY27 cells (Δ*cheY*) (Wang et al., 2018) that were complemented with a fliC^st^ gene (Sowa et al., 2005) that encoded the sticky flagellar filament and lacking the *cheY* gene (encoding a chemotaxis response regulator that switched the flagellar rotation direction) to result in flagella always rotating in the CCW direction, were chosen as the host cell to assess the role of those YcgR variants. The JY27 cells (Δ*cheY*), those derivative cells lacking *yhjH* (Δ*cheY*Δ*yhjH*, referred to as RW1), those lacking *yhjH*/*ycgR* genes (Δ*cheY*Δ*yhjH*Δ*ycgR*, referred as RW3) and the RW3 cells complemented with YcgR (Δ*cheY*Δ*yhjH*Δ*ycgR*::*ycgR*) were firstly assayed. The flagellar rotation speeds of JY27, RW1 (Δ*cheY*Δ*yhjH*), RW3 (Δ*cheY*Δ*yhjH*Δ*ycgR*), and RW3 cells complemented with YcgR (Δ*cheY*Δ*yhjH*Δ*ycgR*::*ycgR*) are 28.3 ± 8.1, 13.1 ± 3.8, 28.5 ± 8.8, and 14.7 ± 4.1 rps, respectively (Figure 4A). The results supported the previous conclusion that the deletion of *yhjH* inhibits the flagellar rotation, and the deletion of *ycgR* relieves the motility inhibition caused by the *yhjH* deletion. Four YcgR variants were then assayed, including D54A and Q38A/D54A/N62A (QDN/AAA) involved in FliG binding in this study, and F117A and E232A that were identified to involve in MotA binding in the previous study (Hou et al., 2020). The flagellar rotation speeds of RW3 cells complemented with the YcgR variants D54A, QDN/AAA, F117A, or E232A are 29.9 ± 7.8, 32.4 ± 10.0, 36.7 ± 8.2 and 32.4 ± 10.9 rps, respectively (Figure 4A), indicating that the YcgR mutants with the weakened YcgR-FliG or YcgR-MotA interaction restored the flagellar speed.

To access the role of YcgR-FliG interaction in the switch of flagellar rotation direction, RW3 cells (Δ*cheY*Δ*yhjH*Δ*ycgR*) complemented with *cheY* gene only or both *cheY,* and *ycgR* variant gene were also assayed. For most cells, the flagellar CCW rotation speeds were almost identical to those of CW ones; therefore, we did not distinguish them in the following paragraph. The flagellar rotation speeds of RW3 cells complemented with *cheY* (Δ*yhjH*Δ*ycgR*Δ*cheY*::*cheY*) and *cheY*-*ycgR* (Δ*yhjH*Δ*ycgR*Δ*cheY*::*cheY*-*ycgR*) are 27.2 ± 7.1 and 13.4 ± 5.6 rps and the time ratios in CCW rotation were 88.6 ± 19.3% and 96.5 ± 13.6%. The CCW ratio of the former was comparable to MG1655 (88.2% CCW and 11.8% CW) and MG1655Δ*yhjH*Δ*ycgR* (89.0% CCW and 11.0% CW) previously reported by Fang and Gomelsky (2010), whereas that of latter was comparable to MG1655Δ*yhjH* (96.9% CCW and 3.1% CW). The flagellar rotation speed of RW3 cells harboring both *cheY* and *ycgR* variants D54A, Q38A/D54A/N62A (QDN/AAA), F117A, and E232A was 30.9 ± 11.0, 33.8 ± 8.9, 33.5 ± 11.3 and 34.2 ± 8.6 rps (Figure 4A), respectively, which also indicated the above observation that the weakened YcgR-FliG or YcgR-MotA interaction relieved the flagellar speed inhibition. The CCW ratio of D54A, Q38A/D54A/N62A (QDN/AAA), F117A, and E232A cells was 83.0 ± 18.4%, 72.1 ± 26.1%, 91.4 ± 16.4%, and 86.6 ± 19.4% (Figure 4B), revealing that the weakened YcgR-FliG interaction or YcgR-MotA interaction both resulted in a less CCW ratio. Therefore, we concluded that the YcgR-MotA and YcgR-FliG interactions were essential for inhibiting the flagellar rotation speed and increasing the CCW ratio.

## Discussion

Bacteria respond to environmental stimuli and rapidly regulate their motility in different post-transcriptional ways. For example, *E. coli* could sense attractants *via* chemoreceptors, decrease the CheA kinase activity, downregulate the CheY phosphorylation level, increase the frequency of flagellar CCW rotation to form the flagellar bundle, and finally drive themselves moving toward the attractant (Parkinson et al., 2015). The key to this motility regulation is that phosphorylated CheY (CheY-P) interacts with the rotor (FliM) to switch the flagellar rotational direction to CW and then decides bacterial swimming or tumbling (Kuo and Koshland, 1987). In *Bacillus subtilis*, a clutch protein EpsE was recruited to separate the stator from the rotor and then involved in the motility regulation (Blair et al., 2008). The motility regulation by YcgR in *E. coli* and related species exemplified another post-transcriptional one. YcgR bound to c-di-GMP, directly interacted with motor proteins, decreased the flagellar rotation speed, and biased the flagellar rotation. Interestingly, MotI, a YcgR homolog in *Bacillus subtilis,* was found to bind to c-di-GMP yet acted as a clutch to separate the stator from the rotor like that EspE did (Subramanian et al., 2017). YcgR functioning differently from its homolog MotI suggested different targets of YcgR and MotI. However, the detail of what proteins YcgR was bound to be controversial, as described above.

Previous studies revealed the interactions between YcgR and motors by pull-down, two-hybrid, FRET, and gel filtration assays (Boehm et al., 2010; Paul et al., 2010; Hou et al., 2020). Those assays could not measure the protein–protein dissociation constants essential to unifying different YcgR working models. ITC and SPR were recruited to assess the motor proteins’ affinities of YcgR, but they did not work. As usual, the protein concentration in the cell for the ITC assay should be in the same order of magnitude as that of *Kd*, and the protein concentration in the syringe should be one order of magnitude higher than that in the cell. After those *Kd*s determined, we found that the protein in the syringe should be larger than 1 mM, which is too higher for protein samples. Here, we used a FRET-based YcgR biosensor to measure the motor proteins’ affinities of YcgR in the presence and absence of c-di-GMP. Our work suggested that two motor proteins, MotA (represented by its cytoplasmic domain in the assay) and FliG, could interact with YcgR *in vitro*. The FRET assay used here only showed us the affinities between YcgR and motor proteins. Some other FRET methods, such as single-molecule FRET, may be recruited further to provide kinetic information about the interaction between YcgR and its targets.

The second question was about the roles of YcgR interacting with the motor proteins. Our previous study identified that the c-di-GMP binding motif RxxxR and the C-tail helix of the PilZ domain consisted of the MotA binding site of YcgR, including F117 and E232 (Hou et al., 2020). This study found that residue D54 from a conserved patch in the YcgR-N domain was essential for FliG binding. We then checked the role of YcgR-FliG and YcgR-MotA interaction *via* mutagenesis assay. To tease out the role of YcgR in the flagellar rotation speed and direction, we first performed a mutagenesis assay in *E. coli* RW3 cells without the *cheY* gene, where the flagella exclusively rotated in the CCW direction. All YcgR variants with weakened MotA or FliG binding abilities resulted in an increased CCW speed. We concluded that the regulations of rotation speed were involved in both MotA and FliG. The rotation speed regulation was noted to occur in those cells that could not switch the flagellar rotation direction after the deletion of *cheY*. It was suggested that the regulation of speed and direction might not be directly related, though they may occur sequentially (Nieto et al., 2019).

We next assayed the flagellar rotation speed and CCW ratio in RW3 cells harboring *cheY* and *ycgR* genes. The YcgR variants with the weakened YcgR-MotAc or YcgR-FliG interaction increased the flagellar rotation speed and decreased the CCW ratio. We concluded that the MotA and FliG binding was required for YcgR to inhibit the flagellar speed and increase the CCW ratio. Interestingly, MotA was involved in regulating flagellar rotation direction under this scenario. Considering that no other evidence supported MotA directly handling the switch of flagellar rotation direction and that the FliG-CheY interaction was reported as a key for the direction switch, this regulation might be undertaken *via* the MotA-YcgR-FliG interaction. We suggested that YcgR regulated the flagellar rotation speed and direction *via* interaction with MotA and FliG.

The next question should be the molecular mechanisms of how YcgR regulated the flagellar rotation speed and direction. The MotA-YcgR interaction might impair the torque generated from the interaction of the stator protein MotA and rotor protein FliG, resulting in a low flagellar rotation speed. Besides, the potential YcgR-MotA-FliG interaction was hypothesized to stabilize the conformation of FliG, including the conserved Gly-Gly linker that functioned as a hinge during CW/CCW switching (Brown et al., 2002), increase the resistance between the rotor and the stators, prevent the frequent CW/CCW switch, and also weaken the CW/CCW control ability of phosphorylated CheY-bound FliM. As a consequence, the CCW ratio increased. Further, determining the MotAB-YcgR complex structure or YcgR-FliG complex structure would help answer that question. In recent years, the structures of MotAB complexes from different species have been determined by cryo-EM (Deme et al., 2020; Santiveri et al., 2020). Those studies implied the possibility of determining the MotAB-YcgR complex structure.

**Figure 5:**
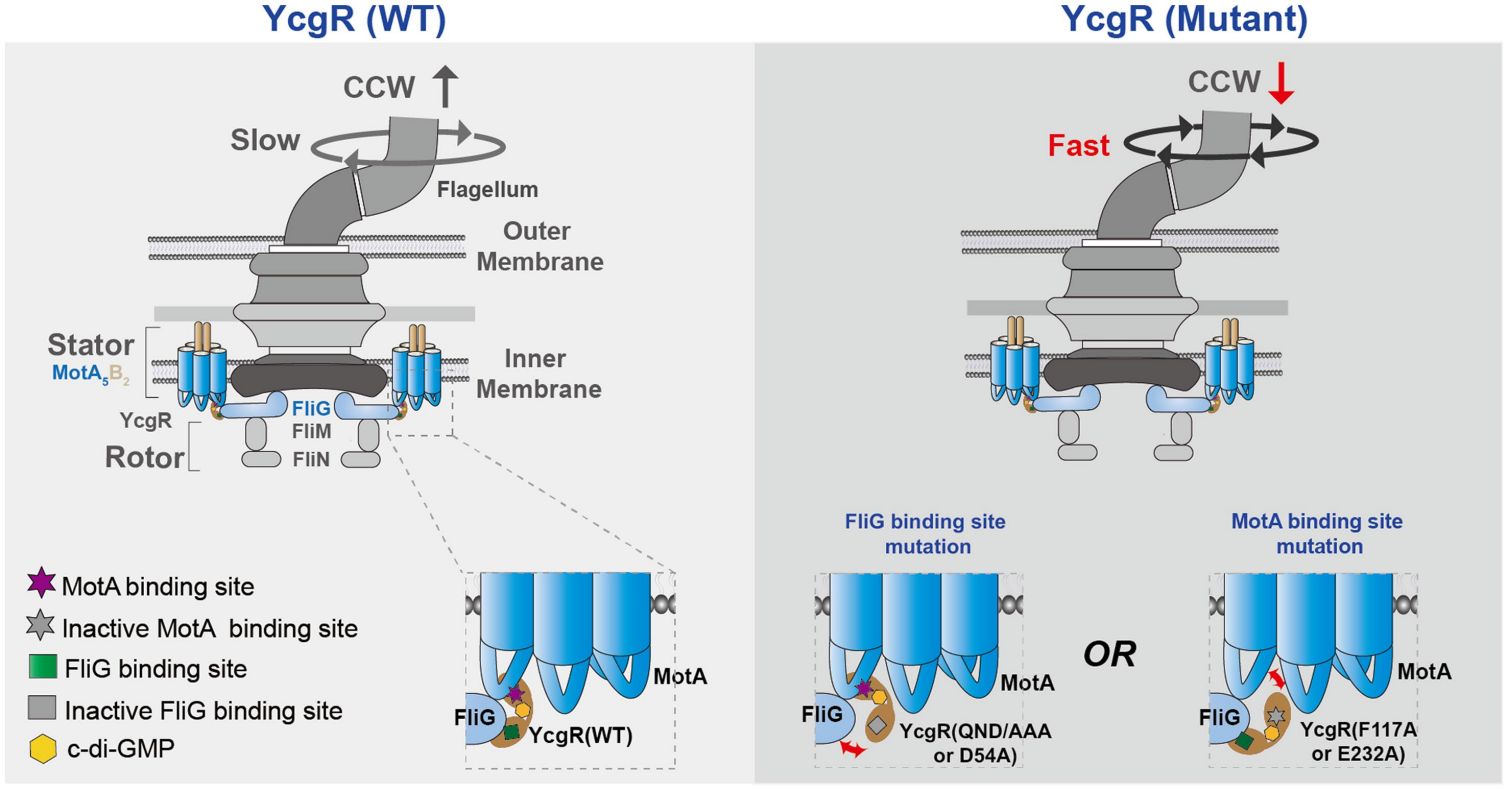
A proposed model illustrates the mechanism of how YcgR works. The bacterial flagella motor was shown in a schematic diagram. MotA interacted with FliG *via* the electrostatic interaction, which generate the torque to drive the flagellar rotation. After being activated by c-di-GMP, YcgR interacted with MotA and FliG to inhibit the flagellar rotation speed and cause a CCW bias. Once the interactions between YcgR and MotA or FliG were weakened by the mutation of MotA or FliG binding residues, the speed was restored, and the CCW ratio decreased.

In conclusion, c-di-GMP-bound YcgR was proposed to bind at the interface of MotA and FliG and interacted with both of them to regulate flagellar rotation speed and direction in the presence of elevated concertation of c-di-GMP (Figure 5). The YcgR variants with weakened MotA or FliG affinities were proposed to dissociate MotA-FliG-YcgR interaction, restore the flagellar output, and decrease the CCW ratio. Though more evidence is required to clarify how YcgR interacted with MotA and FliG, we found that both YcgR-MotA and YcgR-FliG interactions were exactly involved in decreasing the flagellar rotation speed and biasing the rotation direction in the presence of elevated c-di-GMP concentration. Our work provided more knowledge to solve the puzzle of how YcgR regulated flagellar swimming.

## Experimental procedures

### Strains construction and cell cultivation

Strains and plasmids are listed in Supplementary Table S1. The genes of YcgR, MotAc, and FliG were cloned from stain *E. coli* MG1655. The construction of plasmids expressing YcgR, MotAc (residues 70–170 of MotA), FliG, and the complex of FliM and FliN were carried out the same as that in our previous study (Hou et al., 2020). To express the YcgR-based FRET biosensor, the gene of YcgR was cloned into the engineered pET15b plasmid containing an N-terminal 6xHis-tag, monomeric mutation YPet (A206K) and CyPet (A206K) and a multiple cloning site between the FRET pair as previously described (Ohashi et al., 2007; Christen et al., 2010). Strains JY27 (Δ*fliC* Δ*cheY*), RW1 (Δ*fliC* Δ*cheY* Δ*yhjH*), and RW3 (Δ*fliC* Δ*cheY* Δ*yhjH* Δ*ycgR*) were complemented by the plasmid pKAF131 constitutively expresses FliC^st^ (Wang et al., 2018). The genes of YcgR and variants D54A, F117A, E232A, and Q38AD54AN62A (QDN/AAA) were cloned, digested with *Kpn*I and *Sac*I restriction enzymes, and ligated into the pBBRMCS2 plasmid. To obtain the plasmid containing *ycgR* and *cheY*, the *cheY* gene was cloned into the plasmid containing *ycgR* gene as an independent open reading frame located at the immediate 3′-end of *ycgR* with a space sequence “CAGGAGTGTGAA.” Those plasmids were then transferred into RW3 cells to obtain RW3::*ycgR* variant cells and RW3::*cheY*-*ycgR* variant cells. Strains used for bead assay were grown at 37°C in LB medium with the appropriate antibiotics (50 μg/ml chloramphenicol and 50 μg/ml kanamycin). The cells were harvested after the OD_600_ reached 0.5 and 0.6.

### Protein expression and purification

His-tagged MotAc, FliG, mYPet-YcgR-mCyPet (YcgR-biosensor), and mYPet-YcgR-mutants-mCyPet (mutant biosensor) were overexpressed in *E. coli* BL21 (DE3) cells. Transformed cells were cultured to OD_600_ 0.8–1.0 in Luria-Bertani (LB) media at 37°C, and then 0.1 mM (YcgR-biosensor and its mutants) or 0.3 mM (MotAc and FliG) isopropyl β-D-1-thiogalactopyranoside (IPTG) was added. After another 16 h incubation at 16°C, the cells were harvested by centrifugation for the following protein purification. The proteins were purified following the method described by Hou and stored in SEC buffer (20 mM HEPES, pH 7.5, 150 mM NaCl, and 10% glycerol; Hou et al., 2020).

### Binding studies by FRET

Fluorescence measurements were performed with a Hitachi F-7000 spectrophotometer (Hitachi, Japan) at 25°C in SEC buffer. 1.5 μM purified YcgR-FRET or its mutants with increasing concentrations of motor proteins (0–500 μM) were mixed and measured in 1 min. They were excited at 425 nm, and emission spectra were recorded from 450 to 600 nm at 1 nm intervals using slit widths of 5 nm for excitation and emission. The FRET/CFP ratios were calculated by the peak values of CFP (mCyPet, ~480 nm) and YFP (mYPet, ~527 nm) and fitted into a nonlinear logistic equation against motor protein concentrations using Origin 8.0 to determine the dissociation constant *K*_d._ The experiments were repeated three times, and the representative examples were shown.

### Bead assay

Cells were sheared to truncate flagella by passing 40 times between syringes equipped with a 23-gauge needle and harvested by centrifugation at 4,000*g* for 1 min. Then they were washed twice with bead assay motility medium [10 mM potassium phosphate, 0.1 mM ethylenediaminetetraacetic acid, 10 mM lactic acid, and 67 mM NaCl, 1 μM L-methionine (pH 7.0)] and resuspended in motility medium. Coverslips were coated with poly-L-lysine, and a chamber was formed with two pieces of double-sided tape spaced between the coverslip and a glass slide. To measure the motor rotating, 40 μl sheared cells were placed on the glass coverslip coated with poly-L-lysine (Hailun, 188105) and allowed to stand for 1 min, then 1.1 μm-diameter polystyrene latex beads (Sigma, MKCG8400) were attached to the sheared flagellar stubs, incubated for 3 min, and rinsed with 2 ml motility medium. The polystyrene beads were observed by phase-contrast microscopy. Phase-contrast images were recorded at frame rates of 800 fps using a scientific complementary metal-oxide semiconductor (CMOS) high-resolution camera (DCC1545M, United States). Each flagellum was recorded for 50 s. Data analysis was carried out using custom scripts in MATLAB (The MathWorks, Natick, MA). The reported speeds and CCW ratios were averages of those assayed flagella.

### Sequence alignment

The sequence alignment was performed using the Clustal Omega server (Madeira et al., 2019) and displayed using ESPript (Robert and Gouet, 2014).

## Data availability statement

The original contributions presented in the study are included in the article/Supplementary material, further inquiries can be directed to the corresponding authors.

## Author contributions

D-FL and Y-JH designed the project. QH, S-FW, X-XQ, and Y-FS carried out the experiments. D-FL, Y-JH, QH, S-FW, X-XQ, LG, Y-FS, RH, and J-HY contributed to the discussion of results, writing of the manuscript and preparing the tables and figures. All authors contributed to the article and approved the submitted version.

## Funding

This work was supported by grants from National Natural Science Foundation of China (92051101 and 31870037 to D-FL), the National Key R&D Program of China (2019YFA0905500 to D-FL), and the program Youth Innovation Promotion Association CAS (2014079).

## Conflict of interest

The authors declare that the research was conducted in the absence of any commercial or financial relationships that could be construed as a potential conflict of interest.

## Publisher’s note

All claims expressed in this article are solely those of the authors and do not necessarily represent those of their affiliated organizations, or those of the publisher, the editors and the reviewers. Any product that may be evaluated in this article, or claim that may be made by its manufacturer, is not guaranteed or endorsed by the publisher.

## Supplementary material

The Supplementary material for this article can be found online at: https://www.frontiersin.org/articles/10.3389/fmicb.2023.1159974/full#supplementary-material
