# Supplementary material for: Flagellar brake protein YcgR interacts with motor proteins MotA and FliG to regulate the flagellar rotation speed and direction
Source: Front Microbiol. 2023 Apr 14;14:1159974. doi: 10.3389/fmicb.2023.1159974 (PMC10140304; doi:10.3389/fmicb.2023.1159974)
Supplement: Supplementary file 1 [file Data_Sheet_1.docx]

**Supporting information**

**Flagellar brake protein YcgR interacts with motor proteins MotA and FliG to regulate the flagellar rotation speed and direction**

Qun Han ^1, 2#^, Shao-Feng Wang^1, 2#^, Xin-Xin Qian ^1^, Lu Guo^1^, Yi-Feng Shi ^1^, Rui He^3^, Junhua Yuan^3^, Yan-Jie Hou ^4^*, De-Feng Li ^1, 2^ *

^1^ State Key Laboratory of Microbial Resources, Institute of Microbiology, Chinese Academy of Sciences, Beijing, China.

^2^ School of Life Sciences, University of Chinese Academy of Sciences, Beijing, China.

^3^ Hefei National Laboratory for Physical Sciences at the Microscale and Department of Physics, University of Science and Technology of China, Hefei, Anhui, China

^4^ National Laboratory of Biomacromolecules, CAS Center for Excellence in Biomacromolecules, Institute of Biophysics, Chinese Academy of Sciences, Beijing, China.

*Correspondence to: De-Feng Li (lidefeng@im.ac.cn) or Yan-Jie Hou (houyanjie@moon.ibp.ac.cn)

^†^ These authors contributed equally to this work.

**This Supplementary Information file includes the following:**

Figure S1

Figure S2

Table S1

References for table SI reference citations

Figure S1.


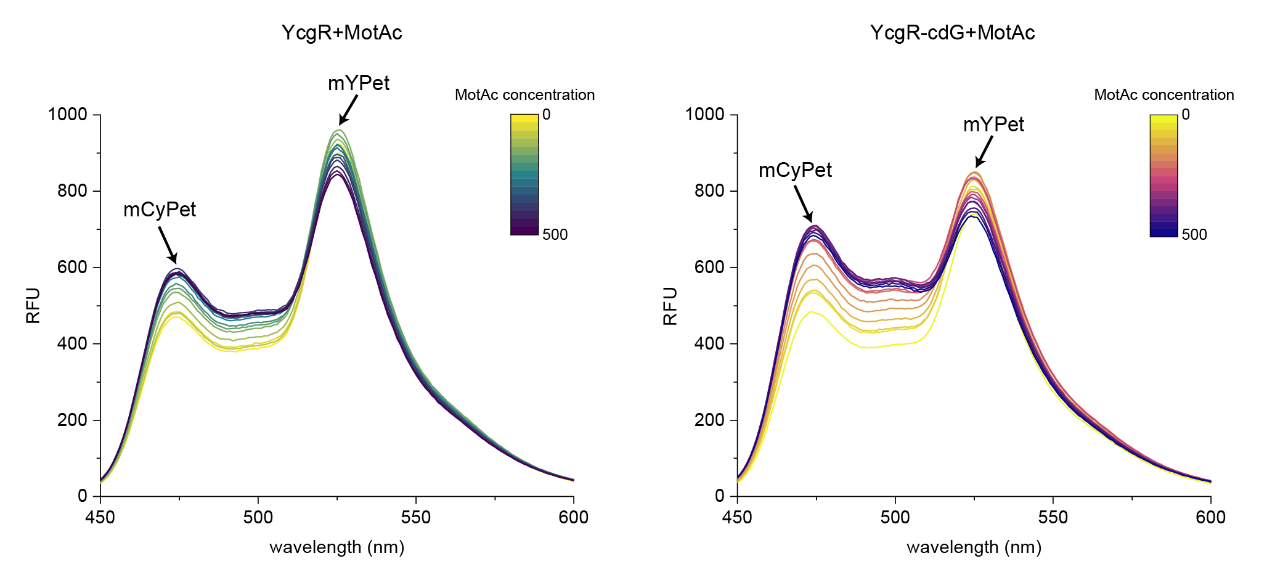


Figure S1. Fluorescence emission spectra of the YcgR-based biosensor. The assays of YcgR-MotAc interaction and c-di-GMP-bound YcgR-MotAc interaction were shown in the left and right panels, respectively. mCyPet and mYPet indicated the peak of CFP and YFP emission spectra, respectively. cdG, c-di-GMP.

Figure S2


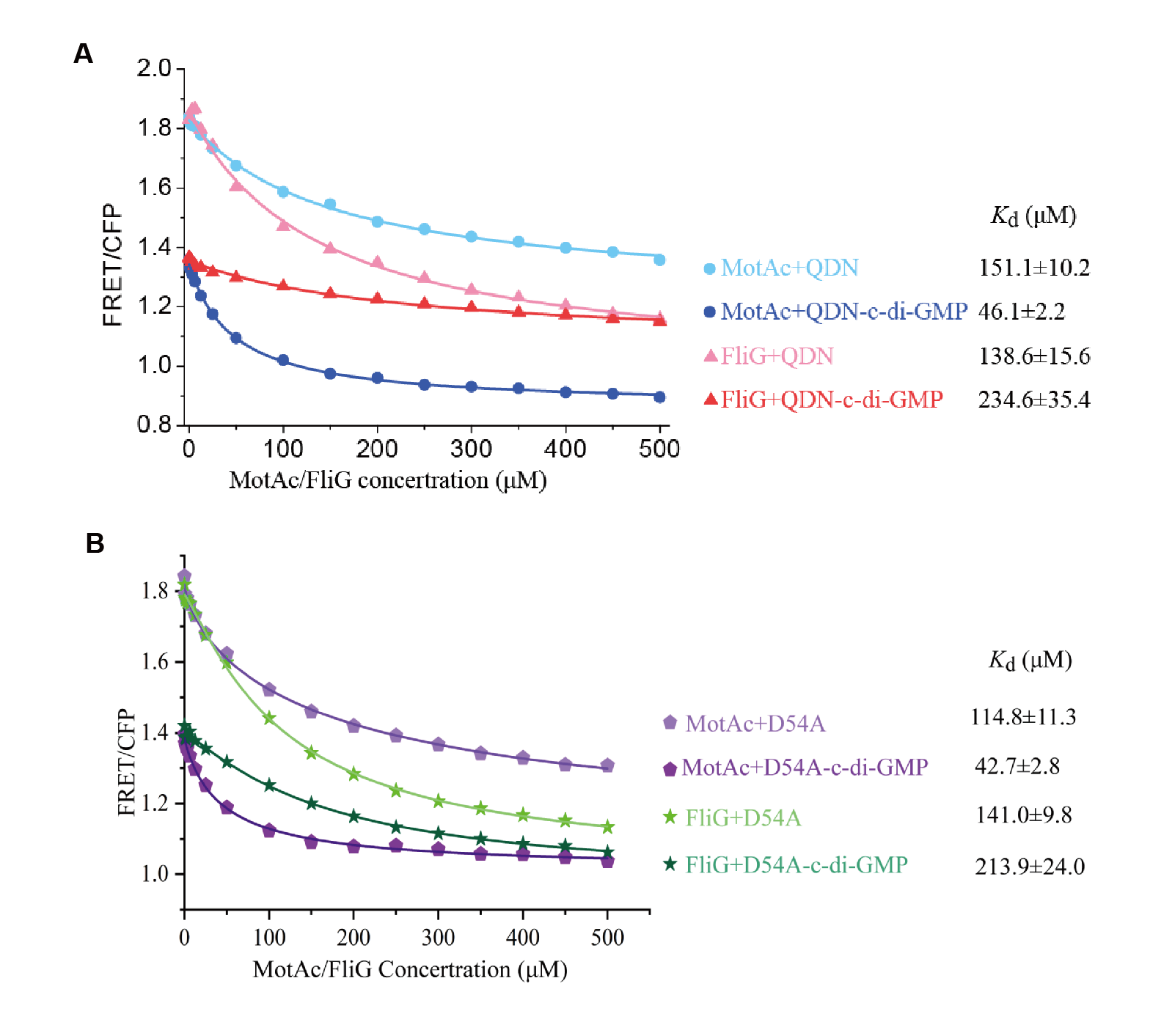


Figure S2. The motor proteins’ affinities of YcgR mutants. The FRET/CFP ratio of YcgR-biosensor mutants, Q38A/D54A/N62A (QDN, A) and D54A (B), and the increasing concentration of motor proteins, are shown. The *Kd* (μM) between mutants and motor proteins are calculated accordingly on the right.

**Table S1.** **Strains and plasmids were used in this study.**

| Strain | Relevant genotype | plasmids | Assay | Derive | Antibiotics |
| --- | --- | --- | --- | --- | --- |
| JY27 | *ΔfliC ΔcheY* | pKAF131(*fliC^st^*) | Bead assay | (1) | Cm |
| RW1 | *ΔfliC ΔcheY ΔyhjH* | pKAF131(*fliC^st^*) | Bead assay | (1) | Cm |
| RW3 | *ΔfliC ΔcheY ΔyhjH ΔycgR* | pKAF131(*fliC^st^*) | Bead assay | (1) | Cm |
| RW3::ycgR(D54A) | *ΔfliC ΔcheY ΔyhjH ΔycgR* | pKAF131(*fliC^st^*)  pBBRMCS2-*ycgR*(D54A) | Bead assay | This study | Cm Km |
| RW3::ycgR(Q38AD54AN62A) | *ΔfliCΔ cheY ΔyhjH ΔycgR* | pKAF131(*fliC^st^*)  pBBR1MCS2-*ycgR*(Q38AD54AN62A) | Bead assay | This study | Cm Km |
| RW3::cheY | *ΔfliC ΔcheY ΔyhjH ΔycgR* | pKAF131(*fliC^st^*)  pBBR1MCS2-*cheY* | Bead assay | This study | Cm Km |
| RW3::cheY-ycgR | *ΔfliC ΔcheY ΔyhjH ΔycgR* | pKAF131(*fliC^st^*)  pBBR1MCS2-*ycgR*-*cheY* | Bead assay | This study | Cm Km |
| RW3::cheY-ycgR(D54A) | *ΔfliC ΔcheY ΔyhjH ΔycgR* | pKAF131(*fliC^st^*)  pBBR1MCS2-*ycgR*(D54A)-*cheY* | Bead assay | This study | Cm Km |
| RW3::cheY-ycgR(Q38AD54AN62A) | *ΔfliC ΔcheY ΔyhjH ΔycgR* | pKAF131(*fliC^st^*)  pBBR1MCS2-*ycgR*(Q38AD54AN62A)-*cheY* | Bead assay | This study | Cm Km |
| BL21(DE3):: *motA*^70-170^ | F^-^ *omp*T *hsd*S_B_ (r_B_^-^ m_B_^-^) *gal* *dcm* (DE3) | pET-22b-*motAc*-His_6_ | Protein expression | (2) | Amp |
| BL21(DE3)::*fliG* | F^-^ *omp*T *hsd*S_B_ (r_B_^-^ m_B_^-^) *gal* *dcm* (DE3) | pET-22b-*fliG*-His_6_ | Protein expression | (2) | Amp |
| BL21(DE3)::*ycgR* | F^-^ *omp*T *hsd*S_B_ (r_B_^-^ m_B_^-^) *gal* *dcm* (DE3) | pET15b-FRET-*ycgR* | Protein expression | This study | Amp |
| BL21(DE3)::*ycgR*^QND/AAA^ | F^-^ *omp*T *hsd*S_B_ (r_B_^-^ m_B_^-^) *gal* *dcm* (DE3) | pET15b-FRET-*ycgR*(QDN/AAA^)^ | Protein expression | This study | Amp |
| BL21(DE3)::*ycgR*^D54A^ | F^-^ *omp*T *hsd*S_B_ (r_B_^-^ m_B_^-^) *gal* *dcm* (DE3) | pET15b-FRET-*ycgR*(D54A) | Protein expression | This study | Amp |

**References**

1. Wang R, Wang F, He R, Zhang R, Yuan J. 2018. The Second Messenger c-di-GMP Adjusts Motility and Promotes Surface Aggregation of Bacteria. Biophysical Journal 115:2242-2249.

2. Hou Y-J, Yang W-S, Hong Y, Zhang Y, Wang D-C, Li D-F. 2020. Structural insights into the mechanism of c-di-GMP-bound YcgR regulating flagellar motility in Escherichia coli. Journal of Biological Chemistry 295:808-821.
